# Draft Genome Sequences of *Helicobacter pylori* Strains HPARG63 and HPARG8G, Cultured from Patients with Chronic Gastritis and Gastric Ulcer Disease

**DOI:** 10.1128/genomeA.00700-13

**Published:** 2013-09-05

**Authors:** Rita Inés Armitano, Gerardo Zerbetto De Palma, Mario José Matteo, Santiago Revale, Soledad Romero, Germán Matías Traglia, Mariana Catalano

**Affiliations:** Instituto de Microbiología y Parasitología Médica (IMPAM, UBA-CONICET), Facultad de Medicina, Universidad de Buenos Aires-Consejo Nacional de Investigaciones Científicas y Tecnológicas, Buenos Aires, Argentinaa; Instituto de Agrobiotecnología Rosario (INDEAR)-CONICET, CCT-CONICET, Rosario, Argentinab

## Abstract

*Helicobacter pylori* colonizes the human gastric mucosa, leading to a spectrum of gastric diseases in susceptible populations. Here we announce the draft genome sequences of strains HPARG8G and HPARG63. The data for both genome sequences provide insights regarding the diversity in gene content and rearrangement of the genomic islands commonly harbored by *H. pylori*.

## GENOME ANNOUNCEMENT

*Helicobacter pylori* strain HPARG8G was isolated from a patient with gastric ulcer disease and strain HPARG63 was recovered from a patient with chronic gastritis. Here we report the draft genome sequences of both strains. A total of 785,600 reads with an average length of 435.02 nucleotides, corresponding to 33-fold coverage for strain HPARG8G and 100-fold coverage for HPARG63, were obtained with a 454 GS titanium pyrosequencer. *De novo* assembly was done using 454 Newbler version 2.6, yielding 47 contigs for HPARG8G and 34 contigs for HPARG63. The genomes were annotated using the RAST server (1) and manually curated. Mauve (2) was used to predict the order and orientation of the contigs, and Artemis (3) was employed to glean details of the two genomes in comparison with the closest overall neighbors predicted by RAST.

The *Helicobacter pylori* HPARG8G genome sequence comprises 1,596,552 bp and 1,568 coding sequences (RAST) as well as 36 tRNAs and 2 rRNAs. The average G+C content was 38.98%. The HPARG63 strain genome sequence contains approximately 1,668,716 bp and 1,623 coding sequences and also has 36 tRNAs and 2 rRNAs, with an average G+C content of 38.79%. The *H. pylori* P12 strain was predicted by RAST as one of the closest overall neighbors of HPARG8G and HPARG63. Both genomes displayed a conserved repertoire of housekeeping genes corresponding to various metabolic pathways. The two genomes each showed a complete *cag* pathogenicity island (*cag* PAI), with the complex rearrangement in the region delimited by *dapB* and *murI* genes described in the Mongolian gerbil-colonizing strain *H. pylori* B8 (4). In HPARG8G and HPARG63, the *cagA* gene was located 12,726 bp and 11,982 bp downstream from the *cagB-cag1* region, respectively. Consequently, the complete *cag* PAI gene order might not be as highly conserved as had been proposed previously (5). The HPARG63 strain contained two transposon of plasticity zone (TnPZ) elements (6). TnPZ type 1b lacked the genes HPP12_455 through HPP12_457 found in the same element in strain P12; the HPP12_459 through HPP12_461 genes from the type IV secretion system TFS4 were also absent (7). In both strains, this TnPZ was inserted in a pseudogene similar to *hp0464* of strain 26695 (7). In addition, the HPARG63 element harbored the *virD2-virD4*, *virB11-virB9*, and *topA* genes with the highest homology to genes of other *H. pylori* strains. The second element was a TnPZ type 2 remnant located in an unusual chromosomal insertion site, containing few type IV secretion system TFS3 genes. The strain HPARG8G carried only *xerD*, *jhp0959*, *jhp0949*, and *jhp0948* genes from the TnPZ type 1b left end (6) and *hp0444*, *hp0445*, and *hp0446* genes from the TFS4, inserted in a methyltransferase type II system. Our findings support the hypothesis that although the TnPZs are genomic elements transferable as a whole, they often contain only subsets of the genes present on the complete islands (6, 7).

### Nucleotide sequence accession numbers.

The first versions of the HPARG8G and HPARG63 sequences are accessible at the European Nucleotide Archive (http://www.ebi.ac.uk/ena/) under accession numbers CBKZ010000001 through CBKZ010000047 and CBKY010000001 through CBKY010000034.
